# Supplementary figures and images for: Curcumin improves D-galactose and normal-aging associated memory impairment in mice: In vivo and in silico-based studies
Source: PLoS One. 2022 Jun 29;17(6):e0270123. doi: 10.1371/journal.pone.0270123 (PMC9242463; doi:10.1371/journal.pone.0270123)

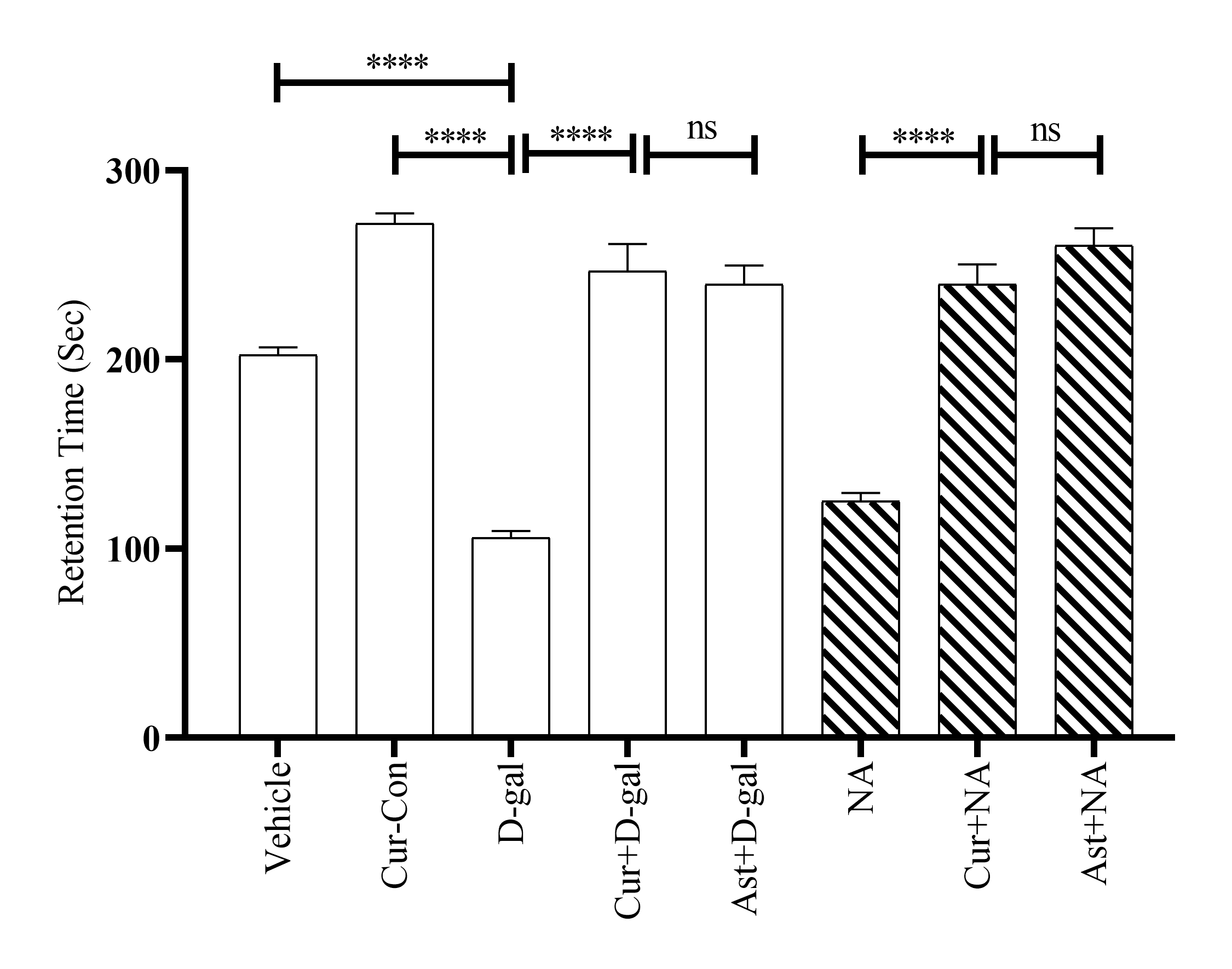

Supplement: S1 Fig — The RT was calculated by performing PA tasks among Vehicle, Cur-Con, D-gal, Curcumin + D-gal, Ast + D-gal, NA, Curcumin + NA, Ast + NA groups. RT was expressed in second. Data was presented as mean ± SEM, n = 8 each group; ****p < 0.0001, ns = not significant. (TIF) [file pone.0270123.s001.tif]
